# A Fuzzy Crew Rostering Model Based on Crew Preferences and Seniorities considering Training Courses: A Robust Optimization Approach

**DOI:** 10.1155/2022/8415169

**Published:** 2022-08-29

**Authors:** Bahareh Shafipour-Omrani, Alireza Rashidi Komijan, Seyed Jafar Sadjadi, Kaveh Khalili-Damghani, Vahidreza Ghezavati

**Affiliations:** ^1^Department of Industrial Engineering, South Tehran Branch, Islamic Azad University, Tehran, Iran; ^2^Department of Industrial Engineering, Firoozkooh Branch, Islamic Azad University, Firoozkooh, Iran; ^3^Department of Industrial Engineering, Iran University of Science and Technology, Tehran, Iran

## Abstract

Crew scheduling problem is divided into crew pairing problem (CPP) and crew rostering problem (CRP). In this paper, a rostering model is presented to assign crew to pairings in such a way that total weighted preference is maximized. Crew members declare which parings they wish to be assigned and which ones are undesirable for them. A score is calculated in the objective function if a crew member is assigned to his/her preferred pairing, and a penalty is considered if he/she is assigned to an undesirable pairing. Moreover, crew seniorities are considered in calculating total preference. In addition, the model considers standard rules and regulations as well as crew attendance at the required training courses. The model is formulated in such a way that inconsistent crew members are not assigned to a flight. Due to the uncertainty in determining of the seniority weight, this parameter is considered as fuzzy. At the end, the robust counterpart of the nominal model is developed due to the uncertainty of time away from the base (TAFB). In this research, the issue of inconsistent crew in rostering problem is considered for the first time. Moreover, a new scoring mechanism is introduced to calculate desirable and undesirable assignments in the objective function. The proposed CRP is solved using the genetic algorithm (GA), and its performance is verified in comparison with GAMS in some test problems. On average, the optimality gap in GA is only 0.5 percent. Finally, the proposed model is examined with real-world data from Air India Airline. In comparison with the previous research studies, the suggested model (scoring mechanism) reduced the number of undesirable rosters by 61.59%.

## 1. Introduction

Using the mathematical models in airline operations is an interest field for researchers. Because of the complexity of optimization problems in the airlines, they are usually divided into several smaller problems: flight scheduling (FS), fleet assignment (FA), aircraft maintenance routing (AMR), and crew scheduling (CS) [1, 2]. Flight scheduling is the starting point of airline operations [3]. Flight scheduling output is utilized as fleet assignment input. The purpose of fleet assignment is to assign fleet types to flights scheduled in the previous step. Following FA problem, individual aircraft routing is planned so that maintenance requirements are satisfied [4].

One of the major topics in airlines is crew scheduling problem (CSP). Due to its complexity, CSP is separated into two phases: crew pairing problem (CPP) and crew rostering problem (CRP) [5]. Pairings are generated in CPP based on carrier rules and regulations [6]. A pairing includes a set of sequential duty days separated by layovers. The purpose of CPP is to cover all flights in such a way that crew cost is minimized. The pairings created in the CPP are assigned to crew in CRP, and each (co)pilot's roster is determined [7]. Although the CPP focuses on cost minimization, the aim of CRP is to maximize crew satisfaction by assigning balanced workload and meeting crew preferences. There are three different approaches in CRP: bidline, personalized with strict seniority, and personalized with global objective [8]. In bidline approach, which was common in North American airlines, anonymous monthly schedules, called rosters, are generated. Then, crew members bid for the rosters according to their preference based on seniority. In a personalized approach with strict seniority, which is an emerging approach in North America, crew preferences are met sequentially in decreasing order of seniority. In personalized approach with global objective, crew assignment is done in such a way that total satisfaction is maximized without considering seniority [9].

Crew rostering problem is one of key the phases of airline scheduling. If crew members are assigned to their favorite rosters, a high degree of satisfactions is resulted. Using mathematical models in rostering problem leads to crew satisfaction. In this paper, crew members are assigned to rosters in such a way that maximum satisfaction is reached. To calculate the satisfaction, a new scoring system is used. On the other hand, the issue of travel safety is one of the most important issues for airlines. Assignment of inconsistent crew to a single flight threatens flight safety considerably. As a main motivation, we develop the proposed model to prevent assignment of inconsonant crew to a single flight.

A new model for CRP is formulated in this paper. The objective function maximizes total weighted preference. Instead of using simple bidline or personalized approach, a new mechanism for calculating preference is used in this paper. At first, each crew member declares if he/she prefers to be assigned to a given pairing or not. If they are assigned to one of their preferred pairings, one positive score is calculated in the objective function. Similarly, a negative score (penalty) is considered if a crew member is assigned to an undesirable pairing. The objective function maximizes crew preferences considering seniority. On the other hand, the model considers a crucial qualitative factor in crew assignment to flights. As some of the crew members may be inconstant and have bad work relations to each other, the model prevents to assign them to a single flight. Assignment of the inconsistent crew to a flight may lead to irrecoverable damage. Moreover, crew attendance at the required training courses and some of the airline regulations such as maximum time away from the base (TAFB) and maximum flying hours are considered in assigning crew to rosters. As TAFB is an uncertain parameter in real-world cases, robust counterpart of the proposed model is formulated using the approach of Bertsimas and Sim [10]. A genetic algorithm is used to solve the model for an Indian airline (Air India). GA efficiency in solving the proposed model is examined by an exact solution obtained by CPLEX in GAMS for a variety of small and medium-size examples. This paper benefits from two main contributions. The first one is using a scoring mechanism instead of bidding for preferred pairings. Against previous research studies, both willingness and unwillingness of crew in assigning to each pairing are considered. The second one is considering the issue of inconsistent crew. This important qualitative factor should be considered to ensure a safe travel for passengers.

The organization of the paper is as follows. The literature on the crew rostering problem is discussed in Section 2. In Section 3, problem statement and mathematical model are described in detail.  Section 4 discusses the solution approach, application of the CRP model in a real-world case study, and the obtained results. Finally, Section 5 concludes with findings and ideas for future studies.

## 2. Literature Review

In this section, a comprehensive review on the CRP is presented. Different research studies have been classified based on if their contributions are in solution approach (heuristic, metaheuristic, and exact) or development of the mathematical model. Although many studies have concentrated on developing solution approaches, only a few ones have contribution in mathematical formulation.

### 2.1. Development of the Solution Approach

Boubaker et al. [11] proposed two heuristic algorithms to solve the CRP. The first approach was the standard branch-and-price algorithm, and the second one was a combination of Elhallaoui et al. [12] with the branch-and-price. Computational findings indicated that the combined method for large-scale problems performed better than standard ones. Maenhout and Vanhoucke [13] considered the use of the additional crew and developed a scatter search algorithm to solve the CSP. The result of comparing the proposed approach with the branch-and-price algorithm and variable neighborhood search demonstrated that this technique could result in high-quality solutions. Saddoune et al. [14] discussed the weaknesses of the traditional approaches, solved the CRP using the dynamic column generation method, and implemented it in an American airline. An integrated approach for aircraft and crew schedule recovery problems was proposed by Zhang et al. [15]. To solve the real problem, they used a two-level metaheuristic algorithm and showed that it could lead to high-quality solutions. Zhang et al. [16] used the Tabu search algorithm to solve the CRP and used the concept of search memory, which accelerated the convergence. By testing the samples, the findings indicated that their method achieves near to optimal solutions in a reasonable time. Doi et al. [17] addressed obtaining a fair roster for the crew by applying a two-level decomposition approach. Zhang et al. [18] developed three multiobjective evolutionary algorithms to search for Pareto solutions. The experimental results on a Chinese airline proved that the proposed technique was capable to improve the rosters' quality by balancing the crew workload. Zhang et al. [19] presented an implicit mathematical model using bidline approach and developed a new variable neighborhood search to solve it. The results indicated that the proposed approach could provide high-quality solutions for two different scenarios of the crew workload. Chutima and Arayikanon [20] formulated a CSP in a low-cost airline and solved it using a multiobjective evolutionary optimization algorithm (MOEA). Comparing the proposed algorithm results with the honeybee mating optimization (HBMO) indicated that MOEA was superior in terms of convergence criteria. The improved discrete particle swarm optimization (IDPSO) method was proposed by Zheng [21] to solve CSP. The findings showed that IDPSO's performance was very efficient. Zhou et al. [22] improved an ant colony algorithm to solve the CRP in a biobjective model optimizing fairness and satisfaction. They proposed a hybrid complementary heuristic strategy and a local search strategy to solve the model adequately. They showed that the novel method led to higher-quality solutions than the greedy algorithm and other prominent multiobjective optimization techniques. Chutima and Krisanaphan [23] considered crew cost minimization as well as balancing the workload of pilots. They solved the proposed model with the adaptive nondominated sorting differential evolution algorithm III (ANSDE-III) and compared the results with the NSGA-III and the multiobjective differential evolutionary approach (MODE). The results showed that ANSDE-III performed better than other algorithms.

### 2.2. Development of CR Models

Chen and Chou [24] formulated pilot roster recovery problem with the aim of minimizing the maximum flight time of each pilot after a disruption and reduced exchange costs. They solved the model with the nondominated sorting genetic algorithm II (NSGA-II) to find Pareto solutions and applied the constraint-loosening mechanism (CLM). The results indicated that the proposed method led to high-quality solutions to the CR problem. Crew changeability in case of delay and disruption was offered by Ilagan and Sy [25]. According to the results, although the proposed model had increased costs, the delay had been significantly reduced. Kasirzadeh et al. [26] formulated the personalized CR problem with undetermined pairings and fixed pilots. The model aimed to optimize crews' preferences and their cost. They used the approach of Saddoune et al. [14] and obtained an acceptable level of crew satisfaction. Armas et al. [27] proposed the CRP with the aim of fair distribution of workload among crew members, and they applied regulations that had not been considered in previous studies. To solve this model, they used the multistart randomized heuristic algorithm and showed that this algorithm has the ability to solve the problem with high quality. An integrated model for CSP was presented by Zeighami and Soumis [8]. They considered pilots' vacation requests in the CP to generate better pairings. The authors formulated the model by minimizing pairing costs and maximizing the desired number of vacations. The experiment results showed that combining Benders' decomposition algorithm and column generation could significantly save CPU time. Quesnel et al. [28] considered rewards for the pairings that matched the crews' preferences in the objective function. They solved the model via column generation and implemented it to a North American airline. The findings demonstrated that the suggested model could improve CRP solutions compared to previous studies. Mirjafari et al. [29] presented an integrated model for aircraft routing and CSP with the aim of minimizing crew, aircraft replacement, maintenance, and deadhead flight costs. They suggest a new frame for maintenance operations based on flight hours. Because the Lagrangian relaxation method results have a lower gap to an optimum solution, this method is better than the particle swarm optimization (PSO) algorithm. A new integrated model for fleet assignment and crew scheduling problems was proposed by Rashidi Komijan et al. [1]. They considered closed routes for crews and fleets simultaneously. To compare the effectiveness of the two methods, PSO and vibration damping optimization (VDO) algorithm solved ten large-scale examples. The findings showed that VDO leads to optimal results in a reasonable time. Saemi et al. [30] suggested an integrated CSP to minimize crew costs. The approach presented a better answer than computing CPP and CRP consecutively. It was also possible to add a day(s) off in a pairing to allow crew members to attend training courses and complete health checks. A multiobjective personalized model for the airline multiskilled was proposed by Baradaran and Hosseinian [31]. The multiskilled crew used in this model was assigned to flights and aircraft due to the crew's experience. They used multiobjective differential evolution (MODE) and nondominated sorting genetic algorithm II (NSGA-II) to analyze the model. According to the results, the suggested model and algorithms could generate adequate schedules for CS problems, according to comparisons between algorithms. Ben Ahmed et al. [32] assigned the aircraft and crew to each flight simultaneously and included maintenance constraints in their model. They solved the robust mixed-integer programming model using the decomposition approach and achieved good results.  Table 1 briefly compares the proposed CRP model with the previous research studies.

To our best knowledge and Table 1, previous research studies mainly focused on cost minimization, fair workload distribution, and use of bidline and personalized approaches for crew rostering. This paper includes two main contributions. The first one is related to the issue of inconsistent (co)-pilots. This critical qualitative component should be evaluated to ensure passenger safety. The model prevents to use inconsistent crew in a single flight. The second contribution is that instead of suggesting preferred pairings, a scoring method is used. In contrast to previous studies, this strategy takes into account both the crew's willingness and unwillingness to assign to each pairing. In other words, one positive score is calculated for each favorable allocation and one negative score is considered for each undesirable allocation in the objective function. Total weighted preference is maximized by considering crew seniority.

## 3. Problem Statement

In this paper, a new mathematical model for the CRP is proposed. The objective function calculates the total weighted preference of the crew. We assume that each crew member declares his/her preferred and undesirable pairings. By assigning a crew member to his/her preferred pairing, a positive score is calculated in the objective function. Similarly, if a crew member is assigned to his/her undesirable pairing, a negative score (penalty) is calculated. The objective function maximizes total weighted preference by considering crew seniority.

In each airline, some of the crew members may have bad relations with each other. Assigning inconsistent crew to a single flight may threaten flight safety. To cover this qualitative factor, the model should prevent to assign inconsistent crew to a flight. Also, several rules and regulations which are common in airlines are considered in the model. Some of these rules are as follows:

- Time away from the base should not exceed a predefined value
- Maximum and minimum flying hours of crew are restricted
- An inexperienced co-pilot must be accompanied by an experienced pilot
- Some of the crew members should attend at training courses

Due to the uncertainty of time away from the base (TAFB), the approach of Bertsimas and Sim [10] is used to formulate the robust counterpart. The advantage of using the approach of Bertsimas and Sim [10] is that the robust model will be linear. Also, the most well-known Indian airline, Air India, is considered as a real-life case. To formulate the model, necessary notations, parameters, and variables are firstly described:

(1) $$\begin{array}{c} Max\sum_{c\in C}\sum_{p\in P}\tilde{w_{c}}{\text{score}}_{cp}-\sum_{c\in C}\sum_{p\in P}\tilde{w_{c}}{\text{penalty}}_{cp}, \end{array}$$

(2) $$\begin{array}{c} \sum_{c\in C_{1}}X_{cp}=1,\;\forall p\in P, \end{array}$$

(3) $$\begin{array}{c} \sum_{c\in C_{2}}X_{cp}=1,\;\forall p\in P, \end{array}$$

(4) $$\begin{array}{c} X_{cp}+X_{c{}^{\prime }p}\leq 1,\;\forall \left(c,c{}^{\prime }\right)\in C_{\text{inc}};\;p\in P, \end{array}$$

(5) $$\begin{array}{c} {\text{TAFB}}_{p}X_{cp}\leq {\text{TAFB}}_{c}^{\max,}\;\forall c\in C;p\in P, \end{array}$$

(6) $$\begin{array}{c} TF_{c}^{\min}\leq \sum_{p\in P}F_{p}X_{cp}\leq TF_{c}^{\max,}\;\forall c\in C, \end{array}$$

(7) $$\begin{array}{c} X_{cp}+X_{cp{}^{\prime }}\leq 0,\;\forall c\in C;\left(p,p{}^{\prime }\right)\in NA, \end{array}$$

(8) $$\begin{array}{c} X_{cp}\leq \sum_{c\prime \in C_{1}^{\prime }}X_{c^{\prime }p},\;\forall c\in C_{2}^{\prime };p\in P, \end{array}$$

(9) $$\begin{array}{c} \sum_{d\in D}Y_{cd}\geq 1,\;\forall c\in C^{\text{inst}}, \end{array}$$

(10) $$\begin{array}{c} \sum_{p\in P_{d}}X_{cp}+Y_{cd}\leq 1,\;\forall c\in C^{\text{inst}};d\in D, \end{array}$$

(11) $$\begin{array}{c} 2{\text{score}}_{cp}\leq {\text{Pref}}_{cp}+X_{cp,}\;\forall c\in C;p\in P, \end{array}$$

(12) $$\begin{array}{c} {\text{penalty}}_{cp}\geq X_{cp}-{\text{Pref}}_{cp,}\;\forall c\in C;p\in P, \end{array}$$

(13) $$\begin{array}{c} X_{cp}\leq {\text{Base}}_{cp,}\;\forall c\in C;p\in P, \end{array}$$

(14) $$\begin{array}{c} X_{cp},Y_{\text{cdp}}:\text{Binary}, \end{array}$$

(15) $$\begin{array}{c} {\text{score}}_{cp},{\text{penalty}}_{cp}\geq 0. \end{array}$$

The objective function maximizes total weighted preference of the crew. The way of calculating score and penalty is described in the constraints. Equations (2) and (3) ensure that each pairing is handled by one pilot and one co-pilot. Constraint (4) prevents assignment of inconsistent crew to a pairing. According to (5), the TAFB for each crew should not exceed the maximum allowed value. Relation (6) indicates that the total flying hours for a crew member should fall in the defined interval. Some pairings cannot be handled sequentially due to some reasons such as the destination of the first one is not the same as the origin of the next one. Constraint (7) prevents the assignment of a crew member to such pairings. Constraint (8) states that in each pairing, an inexperienced co-pilot must be accompanied with an experienced pilot. Relation (9) is about attendance of crew at the training course or meetings. Constraint (10) prevents concurrence of attendance at the training course and handling a pairing. Constraints (11) and (12) show the way of calculating score and penalty, which have been used in objective function. According to (11), one score is calculated if a crew member is assigned to his/her preferred pairing. Similarly, if he/she is assigned to an undesirable pairing, a penalty is considered. Constraint (13) ensures that a crew member can only be assigned to the pairings that origin from his/her home base. Finally, Constraints (14) and (15) indicate decision variable types.

The seniority weight of pilots is in the range [0, 1]. Since the nature of this parameter is fuzzy, the Jimenez et al. [33] method has used to convert this uncertain coefficient in the objective function to definite. For more information, refer the study by Jimenez et al. [33]. For this matter, the triangular fuzzy distribution has chosen because of its simplicity and efficiency. After performing the calculations, the following equation replaces the indefinite objective function (1).

(16) $$\begin{array}{c} Max\sum_{c\in C}\sum_{p\in P}\left(\frac{w_{c}^{p}+2w_{c}^{m}+w_{c}^{o}}{4}\right){\text{score}}_{cp}-\sum_{c\in C}\sum_{p\in P}\left(\frac{w_{c}^{p}+2w_{c}^{m}+w_{c}^{o}}{4}\right){\text{penalty}}_{cp}. \end{array}$$

As TAFB is an uncertain parameter, the robust counterpart of the nominal model is formulated using the approach of Bertsimas and Sim [10]. TAFB includes total flying time, sit times, and layovers. $\bar{{\text{TAFB}}_{p}}$ and $\hat{{\text{TAFB}}_{p}}$ have assumed the nominal value and tolerance of time away from the base for pairing *p*. According to Bertsimas and Sim [10], protection level (Γ) is defined to make the model robustness. In other words, the solution's robustness is regulated by defining a protection level, which specifies a maximum deviation from the nominal TAFB. The main purpose of this parameter is to make the solutions generated workable for all uncertain scenarios. If at most ⌊Γ_*i*_⌋ of technical coefficients of the constraint *i* change in the defined interval, the model will definitely be robust. Also, a technical coefficient can be changed up to(Γ_*i*_ −  ⌊Γ_*i*_⌋) without violating the relation. Moreover, *σ*_*cp*_ and *θ*_*cp*_ are variables defined to formulate robust counterpart.

To formulate robust counterpart, constraint (5) is replaced by the following constraints.

(17) $$\begin{array}{c} \bar{{\text{TAFB}}_{p}}X_{cp}+\Gamma \theta_{cp}+\sigma_{cp}\leq {\text{TAFB}}_{c}^{\max}\;\forall c\in C;p\in P, \end{array}$$

(18) $$\begin{array}{c} \theta_{cp}+\sigma_{cp}\geq {\hat{\text{TAFB}}}_{p}X_{cp}\;\forall c\in C;p\in P, \end{array}$$

(19) $$\begin{array}{c} \theta_{cp}\geq 0,\sigma_{cp}\geq 0\;\forall c\in C;p\in P. \end{array}$$

## 4. Solution Approach

According to NP-hard nature of the crew rostering problem, an appropriate optimization strategy is required [34]. Many rules and policies may deploy for crew rostering with thousands of variables in a real-world problem. Furthermore, the presence of binary variables makes the solution approach more complicated.

Various papers in the literature use genetic algorithm to solve the airline crew scheduling problem. The GA is the second commonly applied solution technique and first metaheuristic approach for the CSP after column generation [35]. One of the benefits of the GA is the ability to manage numerous solution search areas [36]. As a consequence, the genetic algorithm is deployed as solution strategy for this study. In this section, problems are solved by a genetic algorithm in different sizes to evaluate the efficiency of the proposed approach. The GA outcomes are assessed and compared to the CPLEX solver in GAMS. Also, the solution method presented in this work and the components of the algorithm utilized are highlighted in Section 4.1.

### 4.1. Genetic Algorithm

#### 4.1.1. Presentation of Solution

The genetic algorithm uses the concept of chromosome to set parameters that offers a proposed problem solution. In practice, the chromosome is a string of solutions to solve the problem. The proposed chromosome can be designed as a string of discrete variables, binary numbers, and a continuous vector based on type of the problem. As a result, choosing an appropriate display form of the chromosome is an essential aspect of algorithm design. The designed chromosome for the proposed CRP is made up of the two-row vector. The number of columns in this vector is equal to the number of pairings |*P*|. The first and second rows specify the pilot number and the co-pilot number, respectively.

#### 4.1.2. Generating the Initial Solution

**Figure 1:**
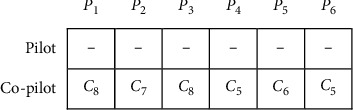
Chromosome with 5 co-pilots and 6 pairings.

In this paper, the co-pilots are randomly assigned to each pairing. Therefore, each compatible co-pilot gets selected to operate in pairing *P*. As an example, 6 pairings, 5 pilots, and 5 co-pilots are available (pilots are numbered from 1–5, and co-pilots are numbered from 6–10). An initial solution for the co-pilots assigned to the pairing is shown in Figure 1. To avoid assigning of a crew member to nonconsecutive pairings, Constraint (7) is applied to prevent the generation of infeasible solutions.

Pilot assignment process begins after all co-pilots get placed in their pairings. Each eligible pilot may get assigned to pairing *P* randomly, if the following conditions met:

1. The pilot should not be in conflict with the co-pilot
2. An inexperienced co-pilot must be accompanied by an experienced pilot in each pairing
3. Set of flights which may be assigned to a specific pilot in a sequence

**Figure 2:**
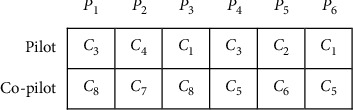
Chromosome with 5 pilots, 5 co-pilots, and 6 pairings.

The pilot row is determined according to Figure 2 for the chromosome.

#### 4.1.3. Generating a New Solution

A suitable operator for generating a random neighborhood solution depends on to the nature of the problem. The CRP is an allocation problem; therefore, we considered the appropriate operators to generate new solutions. These operators are defined in the following section.

*(1) Mutation Operator*. By using the swap operator, two pilots or two co-pilots are selected from two pairings and get swapped if the following conditions are met:

- The co-pilot and the pilot are inconsistent
- If an inexperienced co-pilot is not paired with an experienced pilot
- Pairings that may not be done sequentially by one pilot or co-pilot

**Figure 3:**
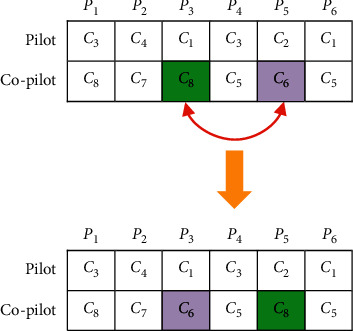
Swap operator.

For the mutation operator, a random number between 0 and 1 is generated. If the number is less than 0.5, a swap operation is performed on the co-pilots, and if it is greater than 0.5, it is performed on the pilots. Swapping the co-pilots of pairings *P*_5_ and *P*_3_ is presented in Figure 3.

**Figure 4:**
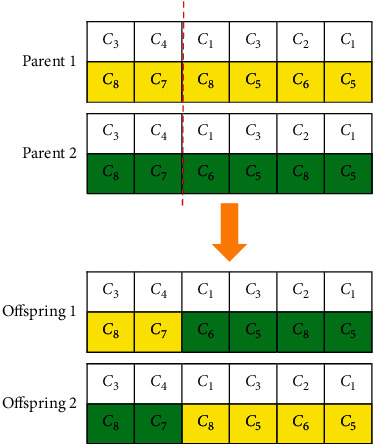
Single crossover.

*(2) Crossover Operator*.  Figure 4 shows the single crossover operator for the CRP. Selecting the pilot/co-pilot row is similar to Section 4.1.3.1 for crossover operator as well.

The proposed chromosome in this paper complies with all constraints of the model except for the maximum/minimum flight hours for crews and maximum time away from the base for crews. To tackle this problem, a penalty function strategy is applied. Therefore, the average violation of these constraints is multiplied by a big number and then subtracted from the objective function.

#### 4.1.4. Genetic Parameter Tuning

In the genetic algorithm, maximum iterations, population size, crossover rate, and mutation rate parameters affect the performance of the algorithm. In this study, the Taguchi [37] is applied to get the best value for mentioned parameters. Each of the GA parameters is considered at three levels, as presented in Table 2.

**Figure 5:**
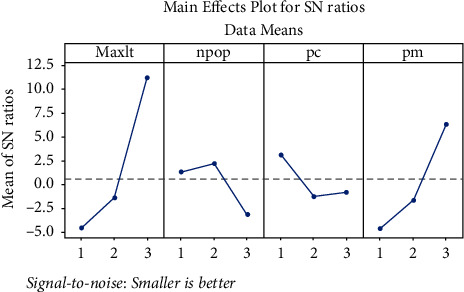
The S/N ratio for the genetic algorithm.

Not all but only part of the factors may reach to their best level in Taguchi's [37] method. Each obtained result from Taguchi [37] method experiment is converting to a “signal-to-noise” ratio. At this rate, the optimal value (average) is called the signal, and the undesirable value (standard deviation) is called the noise. According to the objective function which is to maximize the crew's satisfaction, the higher S/N means more desirability of results. Therefore, the maximum point of the graph is selected for each parameter. Also, the corresponding level is considered as the optimal level. The estimated values for GA parameters using the Taguchi [37] method are illustrated in Table 3 and Figure 5.

### 4.2. Computational Results for Test Problems

**Figure 6:**
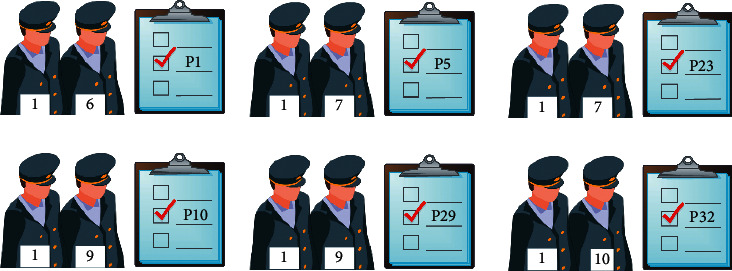
The first pilot's monthly roster.

The model is solved for a real case: Air India airline. Before solving the case study, 30 test problems in different scales are generated and solved using GAMS and GA to justify GA's efficiency in solving the proposed model. This operation is performed on a laptop with 16 GB RAM and Windows 10 64 bit. To illustrate the model results, a problem with 10 crew members is explained in detail. In this example, *C*_1_′, *C*_2_′, and *C*_3_′ are assumed experienced pilots, while *C*_6_′ and *C*_7_′ are inexperienced co-pilots.  Table 4 shows the data for this test problem. By solving the model, crew assignment is done. According to Table 5, one pilot and one co-pilot who are not inconsistent are assigned to each pairing. Crew's rosters can be easily concluded from Table 5. To illustrate, Figure 6 displays the first pilot's monthly roster. As the planning horizon is one month, a crew member may handle several pairings if flight hour constraints are met.

The results of applying the proposed model for 30 numerical examples with different sizes are presented in Table 6. In solving numerical examples by GAMS, the CPU time has been set to 10,800 seconds. According to Table 5, the performance of the GA to solve the proposed model is justified due to two reasons: (1) the maximum optimality gap between the genetic algorithm and exact solution is only 1.482%, and (2) in medium and large-sized problems, the CPU times in the GA are considerably less than GAMS.

**Figure 7:**
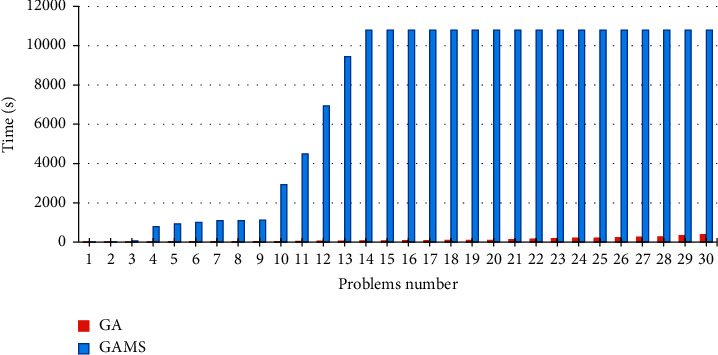
A CPU time comparison between the exact method and GA.

A comparison between the CPU times in GA and GAMS is shown in Figure 7. As maximum solution time has been set to 10,800 seconds in GAMS, computations in problems 14–30 are terminated after three hours.

**Figure 8:**
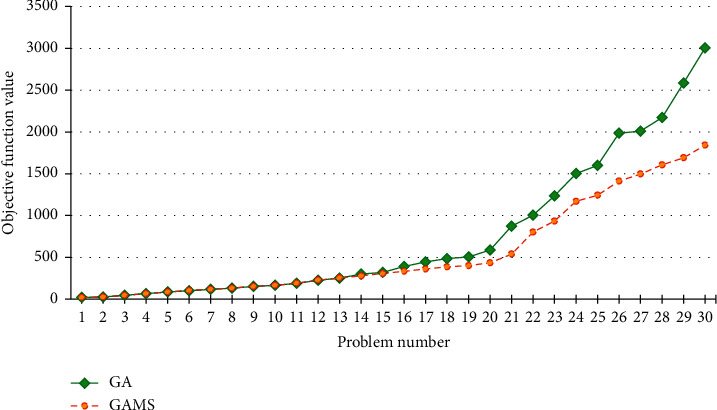
Comparison of the objective function values resulted from GAMS and genetic algorithm.

A comparison of the results between the results of GAMS and genetic algorithm is shown in Figure 8. The results of the objective function are shown in Figure 8. The green and orange points represent the objective function values resulted from GA and GAMS, respectively. It is worth noting that GAMS solution time has been limited to three hours. As a result, GAMS did not reach the optimum solution in problems 14 to 30.

### 4.3. Case Study

Air India is a well-known airline in India, headquartered in New Delhi. As one of the biggest international airlines, Air India has 11% of the domestic market share [38]. Air India's main hub is Indira Gandhi International Airport in New Delhi, with a secondary hub at Chhatrapati Shivaji Maharaj International Airport in Mumbai. According to reports in 2019, Air India fleets include Airbus and Boeing that serve 170 destinations all over the world [32].

**Figure 9:**
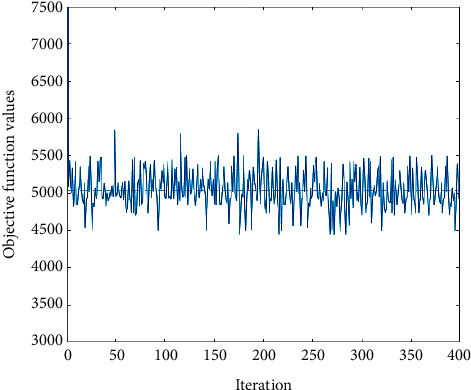
Convergence of the GA results for Air India data.

To solve the proposed model for the case study, a genetic algorithm was applied and the Taguchi [37] approach was used to adjust its parameters. This case included 6190 pairings and 1340 (co)-pilots. The model was solved in 761.188 seconds, and the optimum value of the objective function was 5034.002. The convergence of the GA results is shown in Figure 9. Converging after 400 iterations indicates good solution quality.

As discussed in Section 3, the objective function includes two terms: score and penalty for assigning crew to desirable/undesirable pairings. According to the Air India results, crew members were assigned to their preferred pairings in 72% of assignments.  Table 7 shows the monthly rosters of 20 experienced pilots, and 20 co-pilots as a small sample. Moreover, the attendance days of 90 co-pilots in the required training courses are presented in Table 8.

To compare the performance of the proposed model with the previous research studies in which undesirable assignment penalty had not been considered, penalty term was removed from the objective function and constraint (12) was also disabled. By removing penalty concept, 2065 undesirable pairings are assigned to crews. This means that each crew will have an average of at least two undesirable pairings per month. However, our proposed model has reduced the number of undesirable rosters to 739, which is about 61.59% less than the previous models.

### 4.4. Robust Optimization

In this section, Bertsimas and Sim [10] approach is used to formulate the robust counterpart of the proposed model. The approach of Bertsimas and Sim leads to a linear robust model, while it is independent of knowing the distribution of data and control of the level of protection through parameter Γ [39].

TAFB is subject to several factors such as flight time, flight duration, and connection time, which makes it an uncertain parameter. By considering different values for Γ (0 ≤ Γ ≤ 1), the robust model was solved for numerical example 3, and the results of GAMS and GA were compared. After justifying the performance of the GA, the robust model was applied to the case study and the results were analyzed (Table 9).

**Figure 10:**
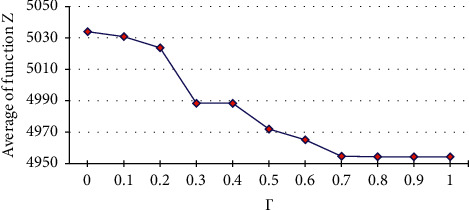
The obtained total weighted desirability from the genetic algorithm for case study.

As represented in Table 9, the optimality gap of GA for numerical example 3 is negligible. Therefore, the performance of GA to solve the robust model is acceptable. According to the robust model applied to the case study, the model acts more conservatively by increasing of the Γ. In the case of Γ=1, it guarantees that feasible solution to be at its maximum value and the objective function to be at its minimum value.  Figure 10 shows the total weighted desirability obtained from the genetic algorithm for 400 iterations.

## 5. Conclusions

Crew scheduling problem is often divided into CPP and CRP and solved consecutively to reduce complexity. In this article, a novel MIP model was introduced for CRP. The difference between the proposed model and previous research is considering a scoring mechanism instead of bidding for preferred pairings, crew seniorities, and inconsistent crew. In addition, the model considers common rules and regulations as well as crew attendance at the required training courses. With this advantage, obviously, better solutions are obtained. Moreover, the robust counterpart of the nominal CRP model was presented to the uncertainty of mission time of the crews (TAFB). In addition, the suggested model's efficiency was assessed using real-world data from Air India Airline. Because the model was NP-hard, the CPLEX solver in GAMS was used for 13 first test problems, and the GA was utilized for 30 test problems with different scales and the case study. The results show that GA has a 1.482% average gap in optimum solution compared to exact solution. The proposed CRP is computed utilizing Air India data, which is one of the largest international carriers. Air India provides services to 170 destinations in 31 countries. The proposed model was tested for 6190 pairings and 1340 crews. For this real-world data, the objective function value was 5034.002 with 761.188 seconds of computational time. This research has two limitations that can be improved in future research studies. The first one is that (co)-pilots' medical checks have not been considered. The second one is that reserve crew have not been included in the model. Moreover, the following suggestions can be considered for future research [40–42]:

- Considering a fair distribution of working time among crew members
- Providing an integrated model for CRP with other steps of the airline scheduling problem, and considering disruption for the crew rostering
- Developing the cooperative game between airlines to exchange crews among them

## Figures and Tables

**Table 1:** Comparison between previous research studies and the proposed model.

Paper	Modeling approach		Solution method	Objective function		Contribution	
	Nonintegrated	Integrated		Scoring mechanism	Others	Model	Solution approach
Saddoune et al. [14]	∗	—	Dynamic column generation	—	Min weighting cost	—	∗
Zhang et al. [15]	—	∗	2-stage heuristic	—	Min cost	—	∗
Chen and Chou [24]	∗	—	Metaheuristic		Minmax flight time	Recovery crew	—
Zhang et al. [16]	∗	—	Metaheuristic	—	Min delay	—	∗
Ilagan and Sy [25]	∗	—	Metaheuristic	—	Min cost	Crew swaps	—
Armas et al. [27]	∗	—	Multistart heuristic	—	Max satisfaction	Work balancing	—
Doi et al. [17]	∗	—	Metaheuristic	—	Min working time deviation	—	∗
Zhang et al. [19]	∗	—	Metaheuristic	—	Min cost	—	∗
Zeighami and Soumis [8]	—	∗	Benders' decomposition and column generation	—	Min weighting cost	Pilots' vacation	—
Mirjafari et al. [29]	—	∗	Lagrangian relaxation	—	Min crew, maintenance, and deadhead costs	Fairly assigning night flights	—
Chutima and Arayikanon [20]	∗	—	Metaheuristic	—	Min repeated flight and max senior pilot preference	—	∗
Zhou et al. [22]	∗	—	Metaheuristic	—	Max satisfaction	—	∗
Rashidi Komijan et al. [1]	—	∗	Metaheuristic	—	Min costs	Times balancing	—
Saemi et al. [30]	—	∗	Metaheuristic	—	Min costs	Considering more days for crew courses and medical checks	—
Baradaran et al. [31]	∗	—	Metaheuristic	—	Max vacations planned and min penalty costs	Multiskilled model	—
Ahmed et al. [32]	—	∗	Benders	—	Min costs	Simultaneously assigning aircraft and crews	—
Chutima et al. [23]	∗	—	Metaheuristic	—	Min costs	—	∗
This paper (2022)	∗	—	Metaheuristic	∗			

**Table 2:** GA parameter levels.

Parameters	Levels
Max iter	200, 300, 400
Pop size	80, 100, 120
CR	0.4, 0.5, 0.6
MR	0.2, 0.3, 0.4

**Table 3:** GA parameter values.

Max iter	Pop size	CR	MR
400	100	0.3	0.4

**Table 4:** Data for 10-crew problem.

Crews		TAFB_*c*_^max^ (hour)	*TF*_*c*_^max^ (hour)	*TF*_*c*_^min^ (hour)	Inconsistent with
Pilots	*C*_1_′	1500	1000	150	*C*_8_
	*C*_2_′	1500	1200	150	—
	*C*_3_′	1500	1200	150	—
	*C*_4_	1500	1200	150	—
	*C*_5_	1500	1200	150	*C*_7_′

Co-pilots	*C*_6_′	1400	1100	100	—
	*C*_7_′	1400	1100	100	*C*_5_
	*C*_8_	1400	1200	100	*C*_1_′
	*C*_9_	1400	1200	100	—
	*C*_10_	1400	1200	100	—

**Table 5:** Crew assignment to pairings.

Pairings	Crews	
	Pilot	Co-pilot
*p*_1_	*C*_1_′	*C*_6_′
*p*_2_	*C*_3_′	*C*_6_′
*p*_3_	*C*_2_′	*C*_7_′
*p*_4_	*C*_3_′	*C*_8_
*p*_5_	*C*_3_′	*C*_7_′
*p*_6_	*C*_4_	*C*_10_
*p*_7_	*C*_4_	*C*_10_
*p*_8_	*C*_5_	*C*_8_
*p*_9_	*C*_3_′	*C*_6_′
*p*_10_	*C*_1_′	*C*_9_
*p*_11_	*C*_5_	*C*_10_
*p*_12_	*C*_4_	*C*_8_
*p*_13_	*C*_3_′	*C*_7_′
*p*_14_	*C*_2_′	*C*_9_
*p*_15_	*C*_5_	*C*_10_
*p*_16_	*C*_3_′	*C*_9_
*p*_17_	*C*_3_′	*C*_6_′
*p*_18_	*C*_5_	*C*_8_
*p*_19_	*C*_3_′	*C*_10_
*p*_20_	*C*_4_	*C*_9_
*p*_21_	*C*_2_′	*C*_10_
*p*_22_	*C*_4_	*C*_8_
*p*_23_	*C*_1_′	*C*_7_′
*p*_24_	*C*_2_′	*C*_7_′
*p*_25_	*C*_5_	*C*_9_
*p*_26_	*C*_2_′	*C*_6_′
*p*_27_	*C*_3_′	*C*_8_
*p*_28_	*C*_4_	*C*_10_
*p*_29_	*C*_1_′	*C*_9_
*p*_30_	*C*_5_	*C*_10_
*p*_31_	*C*_4_	*C*_8_
*p*_32_	*C*_1_′	*C*_10_

**Table 6:** The results of test problems.

Problem no.		No. of pairing	No. of crew	GAMS		GA		Gap (%)
				*Z*	CPU time (s)	*Z*	CPU time (s)	
Small	1	32	10	19.425	1.513	19.425	14.769	0.000
	2	40	14	26.013	3.031	26.013	16.011	0.000
	3	70	18	45.806	62.782	45.806	17.192	0.000
	4	100	22	67.219	785.032	67.219	17.811	0.000
	5	130	26	85.756	921.657	85.744	19.703	0.014
	6	160	30	103.352	1002.140	101.82	20.002	1.482
	7	190	34	118.484	1095.006	117.321	20.892	0.981
	8	220	38	133.105	1101.482	132.008	21.475	0.824
	9	250	42	153.076	1125.015	152.601	23.343	0.310
	10	280	46	165.081	2925.515	165.000	27.107	0.049

Medium	11	350	60	190.380	4500.703	188.234	40.961	1.127
	12	400	66	227.345	6938.796	227.011	46.145	0.146
	13	450	72	253.261	9441.422	252.631	47.412	0.248
	14	500	78	278.205	10800	299.008	59.604	—
	15	550	84	305.829	10800	320.952	63.843	—
	16	600	90	333.307	10800	392.000	68.060	—
	17	650	96	361.161	10800	444.132	71.949	—
	18	700	102	385.513	10800	486.060	84.737	—
	19	750	108	400.192	10800	505.822	98.110	—
	20	800	114	434.377	10800	589.194	98.364	—

Large	21	1000	200	539.373	10800	871.750	133.021	—
	22	1500	250	801.701	10800	1003.108	152.605	—
	23	2000	300	934.006	10800	1236.115	179.881	—
	24	2500	350	1170.196	10800	1501.914	197.001	—
	25	3000	400	1242.201	10800	1600.691	205.514	—
	26	3500	450	1413.135	10800	1985.340	231.109	—
	27	4000	500	1496.866	10800	2010.646	253.002	—
	28	4500	550	1606.530	10800	2174.000	269.836	—
	29	5000	600	1689.977	10800	2586.109	342.168	—
	30	5500	650	1842.583	10800	3004.212	379.617	—

**Table 7:** Monthly rosters for Air India.

Pilots	Pairings	Co-pilots	Pairings
*C*_1_′	*p*_3_-*p*_15_-*p*_410_-*p*_892_-*p*_2001_-*p*_3871_	*C*_791_	*p*_93_-*p*_297_-*p*_305_-*p*_1165_-*p*_1374_
*C*_2_′	*p*_765_-*p*_1380_-*p*_4200_-*p*_4811_-*p*_6152_	*C*_792_	*p*_728_-*p*_923_-*p*_1224_-*p*_2411_-*p*_3005_
*C*_3_′	*p*_1_-*p*_938_-*p*_1778_-*p*_5499_-*p*_6002_-*p*_6112_	*C*_793_	*p*_999_-*p*_1717_-*p*_2060_-*p*_3525_
*C*_4_′	*p*_57_-*p*_21_-*p*_743_-*p*_766_-*p*_1001_	*C*_794_	*p*_14_-*p*_783_-*p*_1183_-*p*_4625_-*p*_5365_
*C*_5_′	*p*_216_-*p*_1230_-*p*_2571_-*p*_4921_-*p*_5101_-*p*_5314_	*C*_795_	*p*_1338_-*p*_3902_-*p*_4382_-*p*_4902_-*p*_6190_
*C*_6_′	*p*_8_-*p*_841_-*p*_3598_-*p*_4009_-*p*_4021_-*p*_4566_	*C*_796_	*p*_210_-*p*_1403_-*p*_3788_-*p*_5582_-*p*_6128_
*C*_7_′	*p*_943_-*p*_2223_-*p*_3467_-*p*_5005_-*p*_5127_-*p*_5128_	*C*_797_	*p*_48_-*p*_655_-*p*_804_-*p*_893_
*C*_8_′	*p*_51_-*p*_394_-*p*_1857_-*p*_2311_-*p*_6188_	*C*_798_	*p*_1195_-*p*_3109_-*p*_5198_-*p*_5727_
*C*_9_′	*p*_1072_-*p*_3725_-*p*_4000_-*p*_4282_-*p*_5920_	*C*_799_	*p*_375_-*p*_831_-*p*_1289_-*p*_3911_-*p*_3927_
*C*_10_′	*p*_790_-*p*_1943_-*p*_2144_-*p*_2756_-*p*_3512_-*p*_6180_	*C*_800_	*p*_202_-*p*_876_-*p*_934_-*p*_1010_-*p*_1256_
*C*_11_′	*p*_21_-*p*_726_-*p*_1451_-*p*_2727_-*p*_5934_-*p*_6107_	*C*_801_	*p*_1689_-*p*_4405_-*p*_4816_-*p*_4970_-*p*_5037_
*C*_12_′	*p*_1191_-*p*_2980_-*p*_3104_-*p*_3788_-*p*_5582_-*p*_6128_	*C*_802_	*p*_234_-*p*_928_-*p*_1440_-*p*_1796_
*C*_13_′	*p*_285_-*p*_961_-*p*_1367_-*p*_3504_-*p*_4597_-*p*_5202_	*C*_803_	*p*_656_-*p*_1275_-*p*_2239_-*p*_3061_-*p*_3400_
*C*_14_′	*p*_1036_-*p*_4436_-*p*_4677_-*p*_4920_-*p*_5135_	*C*_804_	*p*_1008_-*p*_1115_-*p*_1152_-*p*_4636_
*C*_15_′	*p*_30_-*p*_673_-*p*_1558_-*p*_2640_-*p*_3525_-*p*_5555_	*C*_805_	*p*_1261_-*p*_1674_-*p*_1730_-*p*_1954_-*p*_2338_
*C*_16_′	*p*_470_-*p*_1379_-*p*_1776_-*p*_1969_-*p*_1985_-*p*_6161_	*C*_806_	*p*_313_-*p*_630_-*p*_1177_-*p*_2646_-*p*_4789_
*C*_17_′	*p*_1321_-*p*_3040_-*p*_5162_-*p*_5343_-*p*_5691_-*p*_6002_	*C*_807_	*p*_880_-*p*_1326_-*p*_1687_-*p*_2053_-*p*_2188_
*C*_18_′	*p*_811_-*p*_1296_-*p*_2239_-*p*_3061_-*p*_3400_	*C*_808_	*p*_1453_-*p*_2007_-*p*_2396_-*p*_5731_-*p*_5766_
*C*_19_′	*p*_277_-*p*_952_-*p*_3028_-*p*_4816_-*p*_5582_-*p*_6128_	*C*_809_	*p*_934_-*p*_2915_-*p*_3251_-*p*_3256_
*C*_20_′	*p*_500_-*p*_942_-*p*_1101_-*p*_5366_-*p*_5712_-*p*_6001_	*C*_810_	*p*_496_-*p*_1831_-*p*_3140_-*p*_6024_-*p*_6097_

**Table 8:** Attendance in training courses held on different days.

Co-pilots	Training course				
	*d*_1_	*d*_2_	*d*_3_	*d*_4_	*d*_5_
*C*_701_′					×
*C*_702_′					×
*C*_703_′				×	
*C*_704_′	×				
*C*_705_′	×			×	
*C*_706_′		×			
*C*_707_′			×		
*C*_708_′					×
*C*_709_′					×
*C*_710_′					×
*C*_711_′		×			
*C*_712_′	×				
*C*_713_′				×	
*C*_714_′				×	
*C*_715_′				×	
*C*_716_′				×	
*C*_717_′			×		×
*C*_718_′		×			
*C*_719_′	×				
*C*_720_′					×
*C*_721_′			×		
*C*_722_′				×	
*C*_723_′		×			
*C*_724_′		×			
*C*_725_′	×				
*C*_726_′			×		
*C*_727_′		×			
*C*_728_′				×	
*C*_729_′	×				×
*C*_730_′					×
*C*_731_′		×			
*C*_732_′			×		
*C*_733_′					×
*C*_734_′		×			
*C*_735_′	×			×	
*C*_736_′	×				
*C*_737_′		×			
*C*_738_′				×	
*C*_739_′			×		
*C*_740_′				×	
*C*_741_′	×				
*C*_742_′			×		
*C*_743_′					×
*C*_744_′		×		×	
*C*_745_′				×	
*C*_746_′		×			
*C*_747_′					×
*C*_748_′			×		
*C*_749_′					×
*C*_750_′		×			
*C*_751_′	×				
*C*_752_′		×	×		
*C*_753_′				×	
*C*_754_′		×			
*C*_755_′					×
*C*_756_′					×
*C*_757_′	×				
*C*_758_′				×	
*C*_759_′			×		
*C*_760_′			×		
*C*_761_′	×		×		
*C*_762_′				×	
*C*_763_′					×
*C*_764_′	×	×			
*C*_765_′	×				
*C*_766_′		×			
*C*_767_′				×	
*C*_768_′			×		
*C*_769_′			×		×
*C*_770_′					×
*C*_771_′		×			
*C*_772_′			×		
*C*_773_′	×				
*C*_774_′					×
*C*_775_′				×	
*C*_776_′				×	
*C*_777_′		×			
*C*_778_′			×		
*C*_779_′					×
*C*_780_′	×				
*C*_781_′				×	
*C*_782_′			×		
*C*_783_′					×
*C*_784_′		×			
*C*_785_′	×				
*C*_786_′			×		
*C*_787_′			×		
*C*_788_′					×
*C*_789_′		×			
*C*_790_′				×	

**Table 9:** Robust model results.

Case	No. of crew	No. of pairing	⌈	GAMS		GA		GPA (%)
				*Z*	CPU time (U)	z	CPU time (U)	
Numerical example 3 (small-scale)	18	70	0	45.806	63.120	45.806	19.043	0.000
			0.1	45.797	9326.906	45.801	32.152	0.009
			0.2	45.797	4109.891	45.797	37.766	0.000
			0.3	43.815	16977.547	44.221	41.008	0.927
			0.4	43.361	11378.531	43.896	35.423	1.234
			0.5	42.789	6024.208	43.057	28.109	0.626
			0.6	42.229	7466.002	42.612	26.155	0.907
			0.7	42.229	1509.373	42.577	29.000	0.824
			0.8	42.229	3882.041	42.416	29.976	0.443
			0.9	42.229	1557.600	42.416	33.441	0.443
			1	42.229	2493.143	42.416	34.212	0.443

Case study	1340	6190	0	—	—	5034.002	784.006	—
			0.1	—	—	5030.811	797.155	—
			0.2	—	—	5023.722	849.000	—
			0.3	—	—	4988.430	835.637	—
			0.4	—	—	4988.430	802.543	—
			0.5	—	—	4971.959	871.012	—
			0.6	—	—	4965.123	896.424	—
			0.7	—	—	4954.700	903.111	—
			0.8	—	—	4954.361	928.639	—
			0.9	—	—	4954.361	930.007	—
			1	—	—	4954.361	995.295	—

## Data Availability

The data used to support this study are available from the corresponding author upon request.

## Sets and Indices

*P*: Set of all pairings
*p*: Index of pairing (*p* = 1, 2,…)
*C*: Set of all crew members (pilots and co-pilots)
*c*, *c*′: Index of pilots and co-pilots (*c*, *c*′ = 1, 2,…)
*C*_1_: Set of all pilots
*c*_1_: Index of pilots (*c*_1_ = 1, 2,…, *C*_1_)
*C*_2_: Set of all co-pilots
*c*_2_: Index of pilots (*c*_2_ = 1, 2,…, *C*_2_)
*C*_1_′: Set of all experienced pilots
*c*_1_′: Index of experienced pilots (*c*_1_′ = 1, 2,…, *C*_1_′)
*C*_2_′: Set of all inexperienced co-pilots
*c*_2_′: Index of inexperienced co-pilots (*c*_2_′ = 1, 2,…, *C*_2_′)
*C*_inc_: Set of inconsistent crew
*C* ^inst^: Set of crew who must participate at the training course
*P*_*d*_: Set of all pairings that include day *d*
*NA*: Set of all pairings that cannot be performed sequentially by a (co)-pilot
*D*: Set of all days in which the training course is held
*d*: Index of training course (*d* = 1, 2,…)

## Parameters

TAFB_*p*_: Time away from the base of pairing *p*
*TF*_*p*_: Total flying time of pairing *p*
TAFB_*c*_^max^: Maximum TAFB allowed for (co)-pilot *c*
*TF*_*c*_^max^: Maximum flying hours for (co)-pilot *c*
*TF*_*c*_^min^: Minimum flying hours for (co)-pilot *c*
Pref_*cp*_: Binary parameter that equals 1 if pairing *p* is desirable for (co)-pilot *c*, and 0 otherwise
Base_*cp*_: Binary parameter that equals 1 if pairing *p* begins and ends at the home base of (co)-pilot *c*, and 0 otherwise
$\tilde{w_{c}}$: Seniority weight of (co)-pilot *c*

## Decision variables

*X*_*cp*_: Binary variable that equals 1 if pairing *p* is assigned to (co)-pilot *c*, and 0 otherwise
*Y*_*cd*_: Binary variable that equals 1 if (co)-pilot *c* attends at training course held on day *d*, and 0 otherwise
score_*cp*_: Score of assigning (co)-pilot *c* to his/her preferred pairing *p*
penalty_*cp*_: Penalty for assigning (co)-pilot *c* to the undesirable pairing *p*.
